# Heat Shock Proteins in Relation to Heat Stress Tolerance of Creeping Bentgrass at Different N Levels

**DOI:** 10.1371/journal.pone.0102914

**Published:** 2014-07-22

**Authors:** Kehua Wang, Xunzhong Zhang, Mike Goatley, Erik Ervin

**Affiliations:** 1 Department of Grassland Science, College of Animal Science and Technology, China Agricultural University, Beijing, China; 2 Department of Crop and Soil Environmental Sciences, Virginia Polytechnic Institute and State University, Blacksburg, Virginia, United States of America; University of Alabama at Birmingham, United States of America

## Abstract

Heat stress is a primary factor causing summer bentgrass decline. Changes in gene expression at the transcriptional and/or translational level are thought to be a fundamental mechanism in plant response to environmental stresses. Heat stress redirects protein synthesis in higher plants and results in stress protein synthesis, particularly heat shock proteins (HSPs). The goal of this work was to analyze the expression pattern of major HSPs in creeping bentgrass (*Agrostis stolonifera* L.) during different heat stress periods and to study the influence of nitrogen (N) on the HSP expression patterns. A growth chamber study on ‘Penn-A4’ creeping bentgrass subjected to 38/28°C day/night for 50 days, was conducted with four nitrate rates (no N-0, low N-2.5, medium N-7.5, and high N-12.5 kg N ha^−1^) applied biweekly. Visual turfgrass quality (TQ), normalized difference vegetation index (NDVI), photochemical efficiency of photosystem II (Fv/Fm), shoot electrolyte leakage (ShEL), and root viability (RV) were monitored, along with the expression pattern of HSPs. There was no difference in measured parameters between treatments until week seven, except TQ at week five. At week seven, grass at medium N had better TQ, NDVI, and Fv/Fm accompanied by lower ShEL and higher RV, suggesting a major role in improved heat tolerance. All the investigated HSPs (HSP101, HSP90, HSP70, and sHSPs) were up-regulated by heat stress. Their expression patterns indicated cooperation between different HSPs and their roles in bentgrass thermotolerance. In addition, their production seems to be resource dependent. This study could further improve our understanding about how different N levels affect bentgrass thermotolerance.

## Introduction

Heat stress due to increased temperature is a problem in agriculture worldwide. Heat stress induces a series of growth and metabolic responses in higher plants [1], [2], [3]. For example, heat stress redirects protein synthesis in higher plants by decreasing the synthesis of normal proteins accompanied by a dramatic increase in transcription and translation of a new set of proteins: heat shock proteins (HSPs) [4]. Based on their approximate molecular weight, the principal heat shock proteins are grouped into five conserved classes: HSP100, HSP90, HSP70, HSP60, and the small heat-shock proteins (sHSPs, a molecular mass of 15 to 42 kDa identified by denaturing polyacrylamide gel electrophoresis) [4], [5]. HSPs function mainly as molecular chaperones that help other proteins maintain their native conformation, thus improving protein stability under stresses [2]. The role of HSPs to counter act effects of heat stress in plants was first hypothesized based on correlative evidence [6]. There is accumulating evidence that HSPs play important roles in thermotolerance and that some specific HSPs are causally involved in the capacity to acquire thermotolerance. For example: HSP101 in maize (*Zea mays* L.) and *Arabidopsis* [7], [8], HSP90 in *Arabidopsis* [9], HSP70 in tobacco (*Nicotiana tabacum* L.) [10], and sHSPs in maize and creeping bentgrass [11], [12].

Cool-season turfgrasses are subject to high temperature stress during summer in warm climatic regions that often results in quality decline. Some studies have reported that protein changes in response to heat stress or changes in specific HSP expression are related to heat stress tolerance in cool-season turfgrass species, including creeping bentgrass [13]. He and Huang [14] reported the synthesis of several heat-inducible proteins in cytoplasm and membranes of Kentucky bluegrass (*Poa pratensis* L.), and indicated that better heat tolerance in certain cultivars was associated with induction of these proteins during the early phase of heat stress. Park et al. [15] reported that a small heat shock protein (HSP25) was genetically involved in heat tolerance in creeping bentgrass. A later study from the same research group showed that heat sensitivity was associated with reduced capacity of bentgrass variants to accumulate this chloroplastic sHSP[16]. More recently, a proteomic study on *SAG12-ipt* and *HSP-ipt* transgenic creeping bentgrass found that both a plasmid HSP90 and a chloroplast HSP70 were upregulated in *SAG12-ipt* line plants under heat stress [17]. However, there are no specific data existing for the role of other major HSPs, such as HSP101 in heat tolerance of creeping bentgrass. Moreover, how differential nitrogen fertilization rates affect the expression pattern of these major HSPs in creeping bentgrass under stress is unclear, although a study found nitrogen (N) availability influenced HSP production in maize, demonstrated by high-N plants producing greater amounts of mitochondrial HSP60 and chloroplastic HSP24 per unit protein than their low-N counterparts [18].

Nitrogen is the most needed mineral nutrient for plants, and it is also important to maintain good turfgrass quality, including color, density, growth, and resistance to stress conditions[19]. Plants fertilized with N during heat stress had greater fresh and dry weight, and significantly higher membrane thermostability than those fertilized with N before heat stress. This result was suggested to be due to greater rhizospheric N availability during heat stress [20]. A more recent study reported that higher N helped to maintain higher photosynthesis and photosynthetic N-use efficiency in maize under heat stress [21]. In heat stressed cool-season turfgrasses, additional foliar N supply was found to be beneficial [22], [23], with enhanced antioxidative response being suggested as a mechanism accounting for improved tolerance [22]. However, other mechanisms may be important for improved heat stress response by N, such as induction and change of expression pattern of the major HSPs. In addition, although annual N fertilization programs for sand-based creeping bentgrass putting greens are well developed, recommendations for optimum N application during summer heat stress periods are not well defined. For instance, Beard [24] suggested minimizing N application during summer heat stress. He also indicated a need for N to maintain healthy turf, but no specific rates were recommended. Duble [25] also pointed out that very little fertilizer should be used in summer on bentgrass greens with possible monthly applications of N at 12.5 kg ha^−1^.

The objectives of this study were to find optimum N fertilization rate ranges for creeping bentgrass under high temperature and relative humidity conditions that mimic severe summer heat stress, to analyze the pattern of expression of the major members of the HSPs during such periods, and then to study the influence of N on the expression pattern of the HSPs. The N rates chosen in this study were based on a literature search, our previous studies, and the senior author's personal communications with golf course superintendents in Virginia and similar transition zone climates.

## Materials and Methods

### Plant Materials and Treatments

‘Penn A4’ creeping bentgrass was planted in late April, 2009 at 49 kg PLS (pure live seed) ha^−1^ in 19-cm diameter plastic pots(20-cm depth). The pots were filled with gravel 2.0 cm above the bottom with the remaining volume filled with a soil mixture of sand and calcined clay (heat-treated montmorillonite clay mineral, Profile Products, Buffalo Grove, IL) at a volume ratio of 80% to 20% to mimic standard USGA rootzone profiles (USGA 2004). The grass was fertilized with Bulldog brand (28-8-18, 1% ammoniac N, 4.8% nitrate N, and 22.2% urea N; SQM North America, Atlanta, GA) at 5 kg N ha^−1^ every week over the first two months, then reduced to 2.5 kg N ha^−1^ biweekly. Three months after growing under greenhouse mist (20±3/15±2°C, day/night), the grass was moved into a growth chamber. The detailed growth chamber settings were: 38/28°C (day/night), relative humidity 70%/85% (day/night), 450 µmol s^−1^ m^−2^ photosynthetically active radiation (PAR) and a 14-h photoperiod. Grass was hand-clipped to a 12 mm height using an electric shear (3 times a week) throughout the project, except the weeks when grass tissues were sampled.

Foliar spray treatments of N as NO_3_^−^ at 0 (no N), 2.5 (low N), 7.5 (medium N) and 12.5 (high N) kg N ha^−1^ were applied every two weeks (Day 0, 14, 28, and 42) in Hoagland's solution (Epstein and Bloom, 2005) (2.5 mL per pot) with a spray bottle to mimic standard summer application procedures on a golf course putting green. Leaf burning was observed after the first spray at medium and high N rates, particularly the high N rate. Thus all the later N solution applications were followed by an immediate leaf rinse with 100 mL potable water per pot, and no fertilization burn was observed thereafter. A light watering-in with overhead irrigation following liquid fertilizer applications is also a standard summer practice on golf courses. Both KNO_3_ and Ca(NO_3_)_2_ were used as the nitrate sources in the solution. Potassium and calcium levels were equalized across treatments by adding KCl and CaCl_2_ into the lower N treatment solution. Thus, all nutrient levels were the same, except higher Cl^−^ concentration in the lower N treatment solution. A 25-cm plastic pan was placed under each pot, and grass was sub-irrigated with 150 mL potable water per pot daily in the morning to prevent drought stress.

### Sampling and Measurements

Shoots were harvested at Day 1 (one day after initial treatment application plus heat stress), 15, 36, and 50 in the morning. Roots were washed free of soil after the final harvest (Day 50). All samples were immediately frozen with liquid nitrogen and stored at −80°C until analysis, except the portion used for shoot electrolyte leakage and root viability assays.

Turfgrass quality (TQ) was visually rated weekly based on a scale of 1 to9, with 1 indicating poorest or dead turf, and 9 the best possible quality according to Wang and Jiang[26]. Normalized difference vegetation index (NDVI  =  (Infrared_850_-Red_660_)/(Infrared_850_+Red_660_)) and canopy photochemical efficiency of photosystem II (PSII) (Fv/Fm = (Fm_690_−F0_690_)/Fm_690_) were recorded after each TQ reading by using a turf color meter (Fieldscout TCM500, Spectrum Technologies, Plainfield, IL) and a dual wavelength chlorophyll fluorometer (OS-50II, Opti-Sciences, Hudson, NH), respectively.

Shoot electrolyte leakage (ShEL) and root viability (RV) were measured on samples at the last sampling day (Day 50). ShEL was measured according to the method of Blum and Ebercon [27] with modifications [28]. Fresh shoots (100 mg) were excised and cut into 1-cm segments. After being rinsed twice with double deionized H_2_O, shoot segments were placed in test tubes containing 20 mL of double deionized H_2_O. Test tubes were placed on a shaker for 17 to18 h after which initial conductivity (C1) was measured (Conductivity Meter, VWR). Shoot samples were then killed by autoclaving at 121°C for 20 min and conductivity of the solution was re-measured (C2) after the tubes cooled to room temperature. The relative electrolyte leakage was calculated as (C1/C2)*100.

Root viability was determined on whole roots with intact base and tips by measuring dehydrogenase activity with a modified 2,3,5-triphenyltetrazolium chloride (TTC) reduction method [29]. About 300 mg fresh root tissue was cut into 2-cm lengths. Then the root sections were immersed in 15 mL of 0.6% TTC solution (dissolved in 50 mM phosphate buffer plus 0.05% Triton X-100, pH 7.4). The samples were vacuum infiltrated for 5 min to insure infiltration of TTC and then incubated in the dark for 24 h at 30°C. The roots then were drained and rinsed with deionized water twice. Formazan in the roots were extracted with 5 mL of 95% ethanol at 80°C twice and combined extracts were brought to 10 mL. The absorbance of the extract solution was measured at 490 nm with a spectrophotometer (Biomate 3, Thermo Spectronic, Rochester, NY). Root viability was expressed as the absorbance per g fresh weight.

### Protein isolation, SDS PAGE, and Protein gel blot analysis

About 250 mg of liquid nitrogen powdered shoot and root tissues were carefully mixed in a microtube with either 1.5 (shoots) or 1.0 (roots) mL pre-cooled 50 mM Tris-HCl buffer (pH 7.5) containing 2 mM EDTA (ethylenediaminetetraacetic acid), 10% (v/v) glycerin, 1 mM PMSF (phenylmethylsulphonyl fluoride), 1% PVP (polyvinylpyrrolidone) (w/v) and 1 mM DTT (dithiothreitol). The extracts were centrifuged for 20 min at 16,000 g at 4°C, and the supernatant was collected for further analysis. Protein concentration was determined by the method of Bradford (1976). Briefly, 25 µL of protein extract of roots or diluted protein extract of shoots was mixed with 1 mL of Bradford protein reagent (Sigma, USA), and the absorbance was measured at 595 nm after 15 min using a spectrophotometer (Biomate 3, Thermo Spectronic). Bovine serum albumin was used as a standard (Sigma, USA).

Proteins were separated with sodium dodecyl sulfate-polyacrylamidegel electrophoresis (SDS-PAGE) according to the method of Laemmli [30] with some modifications. Protein extract was mixed with same volume of 2× SDS-PAGEsample buffer containing 125 mM Tris-HCl (pH 6.8), 20% (v/v)glycerol, 4% (w/v) SDS, 10% (v/v) β-mercaptoethanol, and0.02% bromophenol blue. An equal amount of protein (40 µg for HSP101, HSP90, HSP70 protein and 30 µg for small HSP) was loaded in each lane. A pre-stained protein standard was loaded on each gel for molecular weight identification. A PROTEIN III electrophoresis unit (Bio-Rad Laboratories, USA) was used to separate the proteins. All the protein extracts were subjected to SDS-PAGE with 5% stacking gel and 10% resolving gel, except small HSPfor which a 12% resolving gel was used. Electrophoresis was performed at 160 V for 50 min at room temperature. The separated proteins were transferred for 1 h at constant volts of 100 and blotted onto 0.25-µm nitrocellulosemembrane (Bio-Rad Laboratories, USA). After blotting, the membranewas blocked with 5% nonfat milk in TBS (25 mM Tris-HCl, 150 mMNaCl, pH 7.5) for 2 h at room temperature. After a brief rinse with TBS, the membrane was incubated in TBS with primary antibodies against HSP101 (Abcam plc., UK), HSP 90 (a kind gift from Dr. Shirasu at University of Tokyo, Japan) [31], HSP70 (Stressgen Biotechnologies), and sHSP (a kind gift from Dr. Heckathorn, University of Toledo, Ohio, USA) [18] ata dilution of 1∶1500, 1∶2500, 1∶1000, and 1∶2000 for 2 h, respectively. Next, the membrane was rinsedin TBS containing 0.1% Tween 20 (TBS-T) 5-min four times and thenplaced for 1.5 h in a solution of either goat anti-rabbit or anti-mouse IgG (secondary antibodies, dilution1∶15,000) conjugated to alkaline phosphatase (Sigma, USA). The membrane was rinsed in TBS-T four times and then developed using a pre-mixture of nitrobluetetrazolium and 5-bromo-4-chloro-3-indolyl phosphate (Sigma, USA). Immunoblotting was conducted for three replications and the representative data are presented here.

### Experimental Design and Statistical Analysis

The experiment was a randomized complete block design with four nitrogen treatments replicated four times. All measurements were analyzed using the samples collected at the sampling days mentioned above. Data were analyzed using PROC GLM (SAS Institute, Version 9.1, Cary, NC). Mean separations were performed using Fisher's–protected Least Significant Difference (LSD) test at a 0.05 significance level, except as otherwise stated herein.

## Results

### Analysis of Variance

Analysis of variance indicated that nitrogen treatments had effects on all the measured parameters at 50 days after heat stress. It also had effects on TQ and NDVI at 1 day after heat stress. Block effect was only observed on Fv/Fm at 1 day after heat stress (Table 1).

**Table 1:** Mean squares from the analysis of variance of turfgrass quality (TQ), normalized difference vegetation index (NDVI),photochemical efficiency (Fv/Fm), shoot electrolyte leakage (ShEL), and root viability (RV) in creeping bentgrass treated with different levels of nitrogen under heat stress.

Day	Source	df	TQ	NDVI	Fv/Fm	ShEL	RV
1	Block	3	0.0040	0.0004	0.0012*		
	Nitrogen	3	0.9740**	0.0069*	0.0002		
	Error	9	0.0323	0.0012	0.0002		
	*R^2^*		0.9099	0.6679	0.6952		
15	Block	3	0.0990	0.0003	0.0057		
	Nitrogen	3	0.1823	0.0006	0.0019		
	Error	9	0.0867	0.0007	0.0096		
	*R^2^*		0.5194	0.6301	0.7256		
36	Block	3	0.0523	0.0032	0.0180		
	Nitrogen	3	0.0612	0.0059	0.0069		
	Error	9	0.0162	0.0043	0.0091		
	*R^2^*		0.7223	0.4157	0.4787		
50	Block	3	0.4769	0.0022	0.0067	33.1975	0.0058
	Nitrogen	3	2.2256**	0.0596**	0.0511**	841.0742**	0.0275**
	Error	9	0.2241	0.0022	0.0029	59.1975	0.0025
	*R^2^*		0.8502	0.9342	0.9067	0.8794	0.8626

**, * mean significant level at *p*<0.01 and 0.05, respectively.

### Turfgrass Quality, NDVI, and Photochemical Efficiency

**Figure 1:**
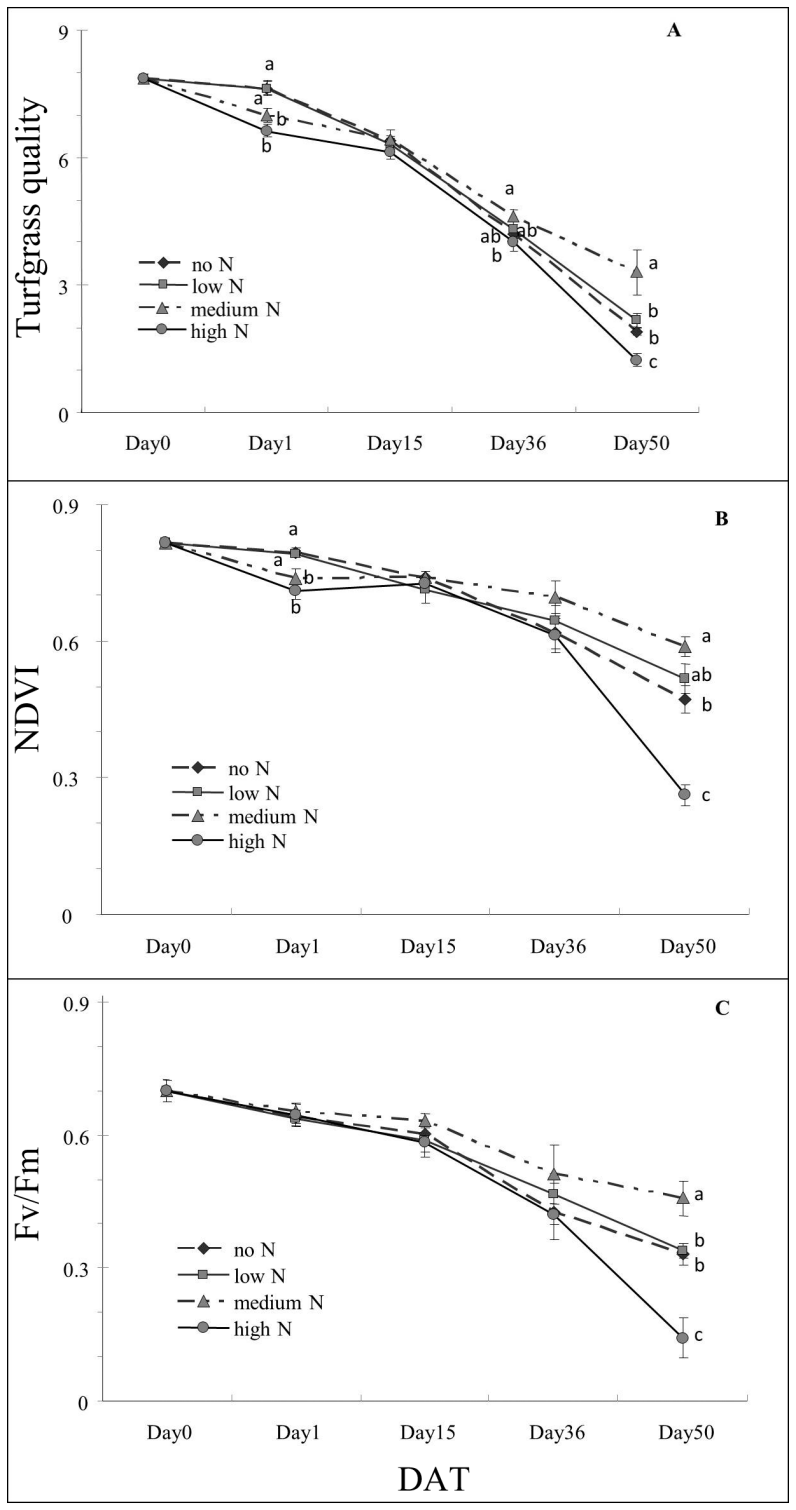
Effects of different N levels on turfgrass quality (TQ) (A), normalized difference vegetation index (NDVI) (B), and photochemical efficiency (Fv/Fm)(C)of creeping bentgrass under heat stress. Means followed by the same letters at each sampling day are not significantly different based on LSD test at *p* = 0.05 level, except TQ (Day 36) at *p* = 0.1 level. Day50: Fifty days after heat stress.

Tufgrass quality (TQ) decreased with the stress regardless of the N level (Fig. 1A). No difference in TQ between N treatments was observed until Day 36 (p<0.1). At this time, grass treated with medium N showed 14% higher TQ than at high N. At Day 50, grass treated with medium N had the highest TQ among the treatments (Fig. 1A). Canopy normalized differential vegetative index (NDVI) and photochemical efficiency (Fv/Fm) followed similar patterns as TQ (Fig. 1B, 1C). Significant differences between treatments were found at Day 50 for both NDVI and Fv/Fm, but not at other sampling dates. Grass at high N had lowest NDVI, which was less than half of that at medium N at Day 50. Grass under medium N showed the highest Fv/Fm readings, which was 38%, 35% and over 200% higher than grass without N, under low N, and high N, respectively.

### Shoot Electrolyte Leakage (ShEL) and Root Viability (RV)

**Figure 2:**
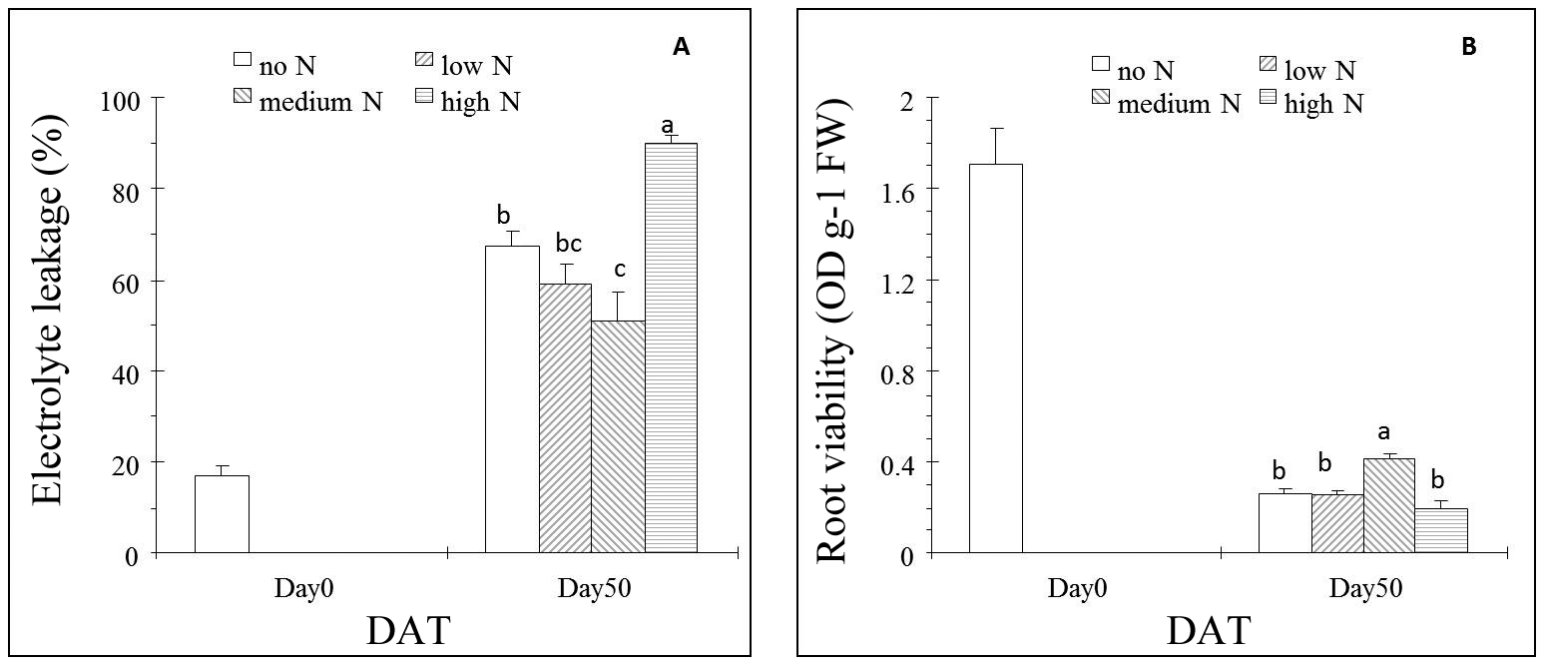
Effects of different N levels on shoot electrolyte leakage (ShEL) (A) and root viability (RV) (B) of creeping bentgrass under heat stress. Means followed by the same letters at each sampling day are not significantly different based on LSD test at *p* = 0.05 level. Day50: Fifty days after heat stress.

Shoot electrolyte leakage increased after 50 d of heat stress regardless of N treatment, simultaneous with decreased root viability (Fig. 2A, 2B). The grass at medium N had lower ShEL than that at both no N and high N, but not the grass at low N. Similarly, the grass at medium N showed higher root viability than the rest.

### Expression of Heat Shock Proteins

**Figure 3:**
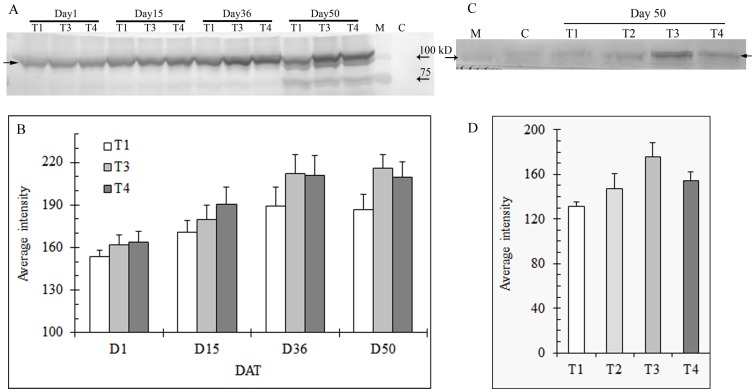
Expression of HSP101 in shoots (A) and roots (C) of heat stressed creeping bentgrass under different N levels using immunoblot and correponding band intensity of HSP101 in shoots (B) and roots (D) using Bio-rad Quantity One software. T1, T2, T3, and T4 represents the treatments of no N, low N, medium N, and high N, respectively. Shoot samples of low N treatment (T2) were omitted in protein gel blot analysis in order to accommodate all the samples across different sampling days on a same gel.M: protein standard for molecular weight; C: sample before heat stress. Equal amounts of protein (40 µg) were loaded to each lane. Solid arrow indicates the HSP, and the open arrow(s) indicate protein standard. Bars indicate standard error of means of different samples in replicate treatments (n = 3).

**Figure 4:**
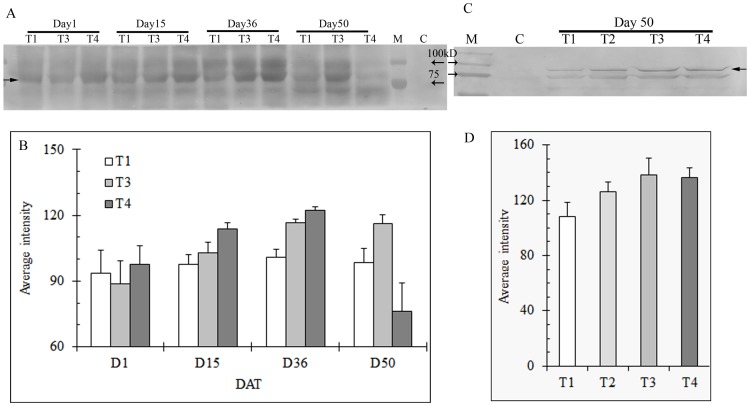
Expression of HSP90 in shoots (A) and roots (C) of heat stressed creeping bentgrass under different N levels using immunoblot and correponding band intensity of HSP90 in shoots (B) and roots (D) using Bio-rad Quantity One software. T1, T2, T3, and T4 represents the treatments of no N, low N, medium N, and high N, respectively. Shoot samples of low N treatment (T2) were omitted in protein gel blot analysis in order to accommodate all the samples across different sampling days on a same gel.M: protein standard for molecular weight; C: sample before heat stress. Equal amounts of protein (40 µg) were loaded to each lane. Solid arrow indicates the HSP, and the open arrow(s) indicate protein standard. Bars indicate standard error of means of different samples in replicate treatments (n = 3).

**Figure 5:**
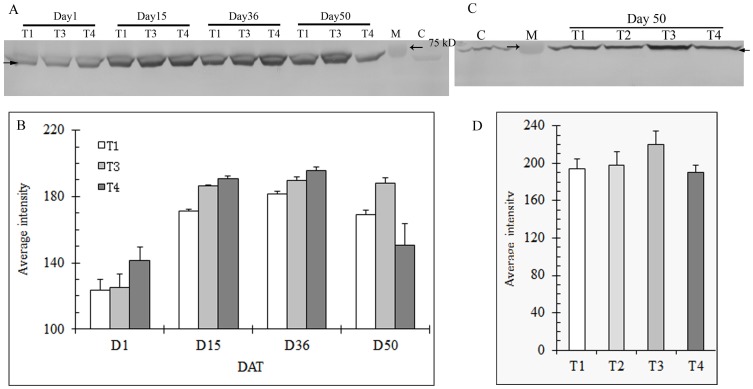
Expression of HSP70 in shoots (A) and roots (C) of heat stressed creeping bentgrass under different N levels using immunoblot and correponding band intensity of HSP70 in shoots (B) and roots (D) using Bio-rad Quantity One software. T1, T2, T3, and T4 represent the treatments of no N, low N, medium N, and high N, respectively. Shoot samples of low N treatment (T2) were omitted in protein gel blot analysis in order to accommodate all the samples across different sampling days on a same gel.M: protein standard for molecular weight; C: sample before heat stress. Equal amounts of protein (40 µg) were loaded to each lane. Solid arrow indicates the HSP, and the open arrow(s) indicate protein standard. Bars indicate standard error of means of different samples in replicate treatments (n = 3).

**Figure 6:**
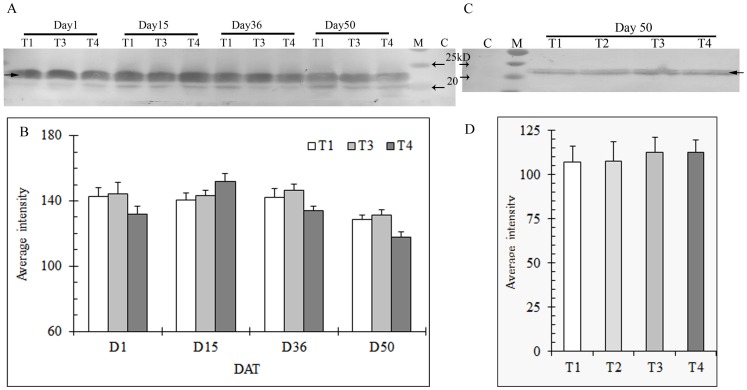
Expression of sHSP in shoots (A) and roots (C) of heat stressed creeping bentgrass under different N levels using immunoblot and correponding band intensity of sHSP in shoots (B) and roots (D) using Bio-rad Quantity One software. T1, T2, T3, and T4 represents the treatments of no N, low N, medium N, and high N, respectively. Shoot samples of low N treatment (T2) were omitted in protein gel blot analysis in order to accommodate all the samples across different sampling days on a same gel.M: protein standard for molecular weight; C: sample before heat stress. Equal amounts of protein (30 µg) were loaded to each lane. Solid arrow indicate the HSP, and the open arrow(s) indicate protein standard. Bars indicate standard error of means of different samples in replicate treatments (n = 3).

Because there was no difference in all the monitored parameters (e.g. TQ, Fv/Fm, and ShEL) between no N and low N treatments (Fig. 1, 2), shoot samples of low N treatment were omitted in protein gel blot analysis in order to accommodate all the samples across different sampling days on a same gel (Fig. 3, 4, 5, 6); otherwise protein gel blot normalization against certain reference proteins would be necessary for a comparison between gel blots.

Protein gel blot analysis showed that HSP101 was induced under heat stress in both shoots and roots of creeping bentgrass. In shoots, a greater amount of HSP101 was present as stress was prolonged. In addition, the grass with higher N generally had more HSP101 in shoots at all sampling dates, except Day 50, when compared to that with lower N. Both roots and shoots at medium N showed a higher level of HSP101 than others at Day 50 (Fig. 3).

Levels of HSP90 in both shoots and roots of creeping bentgrass indicate that it was induced by heat stress. In general, there was a trend that HSP90 at each N level increased with stress until Day 36. In addition, there was a general increase of HSP90 with increased N level at the earlier sampling days (Day 1, 15, and 36). Roots without N had less HSP90 at Day 50 than others (Fig. 4).

HSP70 was present in plants in all treatments before and after heat stress. There was more HSP70 in plants after stress than before stress. Similar to HSP101, the levels of HSP70 increased with stress regardless of treatment within the first five weeks. A general trend of greater HSP70 with increased N level at the earlier sampling days was also observed. In addition, both roots and shoots at medium N showed a higher level of HSP70 than others at Day 50 (Fig. 5).

Like other HSPs investigated here, high temperature induced sHSP in both roots and shoots. Unlike the others, the amount of sHSP did not increase with stress during the first five weeks, with a relatively higher level of sHSP accumulation at higher N treatments only being observed at Day 15 (Fig. 6).

## Discussion

Heat stress affects cool-season turfgrasses negatively. Many studies have reported TQ decline, reduced photochemical efficiency, and other changes under heat stress [29], [32], [33]. As expected, TQ, NDVI and Fv/Fm decreased as heat exposure persisted. The decline of the parameters shown here are unlikely the result of normal growth pattern. In general, these parameters remained relatively stable under optimum temperature condition during experiment periods as reported by Fu and Huang [22] and Xu and Huang [34]. The grass receiving medium N demonstrated positive treatment responses at five weeks of heat stress, and showed higher TQ, NDVI and Fv/Fm than other N treatments at Day 50. Overall, the grass under medium N performed better under stress than at the two lower N levels and at the higher N level (Fig. 1). Nitrogen is an important nutrient for plant growth and development. Proper N availability is also important for plant resistance to stress conditions [19]. Fu and Huang [22] found better TQ and higher Fv/Fm in creeping bentgrass with foliar nitrogen treatment relative to the untreated four weeks after heat stress. Zhao et al. [23] also reported that foliar N fertilization improved photochemical efficiency of heat stressed tall fescue (*Festuca arundinacea* Schreb.). Similar beneficial effects of higher N were also reported in a study with corn under heat [21]. It should be noted the lower TQ and NDVI at Day 1 were due to fertilization burn. We started to rinse the canopy right after fertilization treatment in later applications and no further damage was observed.

In order to further evaluate whether grass under medium N was more heat tolerant, we measured ShEL and RV. Both electrolyte leakage and RV have been widely used to evaluate stress resistance/damage in higher plants [27], [28], [35]. Lower ShEL was observed in grass at medium N concurrently with higher RV at Day 50 (Fig. 2). These data provide further support to our visual and leaf reflectance measurements of better resistance of grass at medium N to long-term heat stress.

Excess N can, however, reduce heat tolerance. In Kentucky bluegrass, plants with high N showed reduced resistance to high temperature [36]. In general, before reaching an optimum N status the stress tolerance of turfgrass increases with an increase of N input and carbohydrate reserves. Excessive N makes the turfgrass less stress tolerant possibly due to excess shoot growth with a cost to carbohydrate reserves [19], [37], [38]. Here we did not monitor the carbohydrate status, but we did find grass at high N performed worst. Similarly, Totten et al. [39] reported in a field study that TQ in summer peaked at 195 kg N ha^−1^per year. Turf quality started to drop at 293 kg N ha^−1^per year, and decreased further at 390 kg N ha^−1^per year. However, their N levels are based on annual rates, and are not specific to a summer heat-stress period, for which their N application rates are not known. Overall, the results indicated the medium N level in this study could be an optimum N rate for managing creeping bentgrass under heat stress.

Heat shock proteins are widely known to play important roles in heat stress tolerance of higher plants [2]. In order to seek the mechanism for the observed better performance of grass at medium N under long-term heat stress, we investigated the expression of several major HSPs, including HSP101, 90, 70, and sHSP.

HSP100 are a family of ATP-binding proteins with chaperone activity to re-solubilize protein aggregates [40], which then can be refolded with the assistance of the HSP70 system [41], [42]. HSP101 proteins have been found in many other grass species, such as rice (*Oryza sativa* L.) [43], [44], wheat (*Triticum aestivum* L.) [45], maize [46], and a perennial grass, *Dichanthelium lanuginosum* (Sw.) [47]. In the study herein we found that HSP101 expression was induced in both roots and shoots of creeping bentgrass under heat stress. In addition, the accumulation of HSP101 protein in shoots seemed to be proportional to stress duration within the first five weeks regardless of N treatments (Fig. 3). Young et al. [46] reported that levels of HSP101 in maize increased in response to heat shock, with abundance depending on different tissues/organs. Al-Niemi and Stout [47] observed HSP101 induction in *Dichanthelium lanuginosum* under both short and long-term heat stress. In maize, HSP101 plays important roles in both induced and basal thermotolerance [8]. HSP101 has also been reported to be a major factor in acquiring thermotolerance in *Arabidopsis* [7]. In the study herein, grass at medium N continued to maintain high levels of HSP101 in both roots and shoots at Day 50, which coincided with better grass performance. Our results indicated that HSP101 could play a role in enhanced resistant to heat stress.

HSP90 is an essential molecular chaperone in eukaryotic cells, with major roles in managing protein folding, protein degradation, and activation of proteins involved in signal transduction and control of the cell cycle [48], [49]. Rutherford and Lindquist [50] proposed a dual involvement of HSP90 in signal transduction and cellular responses to stress, including temperature changes. Some members of the HSP90 family are constitutively expressed, and others are stress inducible [48]. Similar to HSP101, HSP90 in both roots and shoots of creeping bentgrass showed response to heat stress with increased HSP90 protein level along extended stress periods (Fig. 4). An *Arabidopsis* mutant originally identified as deficient in glucosinolate metabolism was found to be thermosensitive due to defective cytosolic HSP90 expression after heat stress. Transient transformation with HSP90 increased its thermostability [9]. However, another study found an HSP90 inhibitor produced in a fungus enhanced *Arabidopsis* thermotolerance [51]. In our study it remains unclear as to whether a relatively high level of HSP90 in shoots at medium N at Day 50 would be related to better overall grass performance. Additionally, the level of HSP90 in roots under high N at Day 50 was still high, although turfgrass quality was low. Considering the possibility that multiple members of HSP90 exist in a single species (e.g., seven were found in *Arabidopsis* [48]), and the fact that HSP90 functions as a capacitor to buffer phenotypic variation in plants [52], further characterization of the role of HSP90 in creeping bentgrass is needed to determine its importance for heat tolerance.

HSP70 proteins are central components of the cellular network of molecular chaperones and are essential for normal cell function [53], [54]. There was basal expression of HSP70 in both roots and shoots of creeping bentgrass before heat stress. After heat stress, an increase over basal levels of HSP70 was observed (Fig.5). Similar results were also reported in a perennial grass, *Dichanthelium lanuginosum* (Sw.), under heat stress [47]. Some members of the HSP70 family are induced by environmental stresses, such as heat or cold. These members are suggested to be involved in refolding and proteolytic degradation of non-native proteins; others are constitutively expressed and referred to as heat-shock cognates [55]. The constitutively expressed form of HSP70 could account for the basal level of HSP70 initially detected under normal temperature. In yeast, it has been shown that HSP70 is required for survival at moderately high temperatures, but not for surviving extreme temperatures [56]. Lee and Schöffl [57] reported that acquisition of thermotolerance was negatively affected in HSP70 antisense *Arabidopsis* plants, accompanied by significantly reduced levels of HSP70/HSC70 proteins. *Arabidopsis* plants overexpressing HSP70 were more tolerant to heat shock [58]. A more recent study found stronger expressions of HSP70 in ‘L-93’ creeping bentgrass than in a relatively less heat-tolerant cultivar, ‘Penncross’ 3 d after heat stress [59]. Like HSP101, the higher level of HSP70 in both shoots and roots at the last sampling day in our study could be important for creeping bentgrass survival of long-term heat stress.

Low molecular weight (LMW) heat shock proteins or small HSPs are the most dominant proteins produced in higher plants upon heat stress [1]. On the basis of their cellular locations, sHSP are placed into 5 classes: cytosolic (class I and II), chloroplastic, mitochondrial, and endoplasmic reticulum related sHSPs [6], [60]. sHSPs play a role as molecular chaperones that bind to partially folded or denatured substrate proteins and thereby prevent irreversible aggregation or promote correct substrate folding to protect cells from stress damage [1]. However, there is no evidence that they are required for normal cellular function [61]. In our study, no sHSP expression was observed in either roots or shoots before heat stress. However, similar to other HSPs analyzed here, they were expressed quickly in response to heat stress (Fig. 6). Zhang et al. [61] confirmed there was no expression of sHSP genes during non-stressed conditions, but a strong activation of this gene in both genotypes of tall fescue under heat stress.

There is accumulating evidence showing that sHSPs are important in plant theromotolerance [11], [62], [63]. For instance, Malik et al. [63] reported significantly higher thermotolerance in carrots (*Daucus carota* L.) overexpressing HSP18.1, and less tolerance to heat shock in plants overexpressing antisense HSP18.1. In creeping bentgrass, a small HSP was reported to be genetically involved in heat tolerance [12], [15]. Heat sensitivity of creeping bentgrass variants was associated with reduced capacity to accumulate chloroplastic small HSPs [16]. Like other HSPs analyzed here, the level of sHSP in shoots was relatively higher under medium N at Day 50, which may confer, at least partially, better grass performance. Heckathorn et al. [18] found a correlation between chloroplast-sHSP production and PSII efficiency, as measured by Fv/Fm. Their later study further indicated that this chloroplast-sHSP plays a direct role in the protection of PSII [11].

As reported above, all the investigated HSPs were up-regulated under heat stress. At the last sampling day, higher levels of all HSPs were observed in shoots or/and roots at medium N. All these data indicate a possible coordination and cooperation between these HSPs. The different classes of HSPs/chaperones are thought to cooperate and play complementary and overlapping roles in protein protection [64]. For example, partially unfolded proteins in the presence of sHSPs can be refolded and reactivated by HSP70 with the participation, in some cases, of HSP100 and HSP60 [64], [65]. Some HSPs/chaperones (HSP70 and HSP90) involved in signal transduction and transcript activation may lead to the synthesis of other HSPs/chaperones [49].

Nitrogen is required in a relatively large amount for biosynthesis of many crucial organic compounds, such as nucleic acids, amino acids, and proteins [66]. Heat stress stimulates a dramatic synthesis of HSPs. Upon heat stress, the fraction of HSPs increases from 1–2% to 4–6% of total cellular proteins [67]. In plants, just class I sHSPs can account for up to 1% of the total protein in cells [68], [69]. Thus production of HSPs involves significant nitrogen and other resource costs [70]. All the investigated HSPs in this study showed an increased accumulation pattern with increased N levels at certain stages during the lengthy heat stress period, such as the levels of shoot HSP70 at Days 1 and 15, which indicated the synthesis of HSPs could be resource dependant. Heckathorn et al. [18] found that high-N plants produced greater amounts of mitochondrial HSP60 and chloroplastic sHSP than their low-N counterparts, suggesting that HSP production involves significant N costs and that N availability influences HSP production in higher plants. However, higher N levels stimulates excess shoot growth with a cost of carbohydrate reserve [19], [37], [38], which could account for the lower accumulation of HSPs in the grass under high N at Day 50. It would be worthy to mention that the induction of HSPs could be due to secondary effects of N treatments instead of just increased availability of nitrogen for protein synthesis, such as the oxidative stress of increased metabolism due to higher N rates [71], [72]. Further study would be warranted.

In summary, medium N (7.5 kg ha^−1^ 14 d^−1^) helped the grass to better survive long-term heat stress. In addition, the expression patterns of the major HSPs suggested they played a role in the improved heat resistance of grass at medium N and that N availability influenced HSP production in grassunder prolonged heat stress. Caution should be taken when making field turfgrass management recommendations based on data from growth chamber experiments. However, our growth chamber research does suggest that a good starting point for future field research would be to apply more than 2.5, but less than 12.5 kg N ha^−1^ every two weeks, when day time high temperatures are between 30 and 40°C.
